# Reconstructing dynamic gene regulatory networks from sample-based transcriptional data

**DOI:** 10.1093/nar/gks860

**Published:** 2012-09-21

**Authors:** Hailong Zhu, R. Shyama Prasad Rao, Tao Zeng, Luonan Chen

**Affiliations:** ^1^Department of Computer Science, Hong Kong Baptist University, Kowloon Tong, Hong Kong and ^2^Key Laboratory of Systems Biology, SIBS-Novo Nordisk Translational Research Centre for PreDiabetes, Shanghai Institutes of Biological Sciences, Chinese Academy of Sciences, Shanghai 200233, China

## Abstract

The current method for reconstructing gene regulatory networks faces a dilemma concerning the study of bio-medical problems. On the one hand, static approaches assume that genes are expressed in a steady state and thus cannot exploit and describe the dynamic patterns of an evolving process. On the other hand, approaches that can describe the dynamical behaviours require time-course data, which are normally not available in many bio-medical studies. To overcome the limitations of both the static and dynamic approaches, we propose a dynamic cascaded method (DCM) to reconstruct dynamic gene networks from sample-based transcriptional data. Our method is based on the intra-stage steady-rate assumption and the continuity assumption, which can properly characterize the dynamic and continuous nature of gene transcription in a biological process. Our simulation study showed that compared with static approaches, the DCM not only can reconstruct dynamical network but also can significantly improve network inference performance. We further applied our method to reconstruct the dynamic gene networks of hepatocellular carcinoma (HCC) progression. The derived HCC networks were verified by functional analysis and network enrichment analysis. Furthermore, it was shown that the modularity and network rewiring in the HCC networks can clearly characterize the dynamic patterns of HCC progression.

## INTRODUCTION

Unravelling the dynamic nature of gene regulation during a biological process is a key challenge in systems biology. The activities of a gene and its functional products reflect the dynamic and integrative influence of its transcription regulators, and other molecules in the signalling pathway (1). The dependencies between these molecular entities are often represented as regulatory relationships in a gene regulatory network (GRN) (1,2), which is normally reconstructed from transcriptional data using a reverse engineering approach (3,4).

The effects of bio-medical interventions on a biological system are normally measured by static (steady-state) or time-course experiments, from which static or dynamic GRNs can be developed. However, many bio-medical studies face a dilemma regarding the use of such an approach. On the one hand, static approaches assume that genes are expressed in a steady state, and hence are not able to exploit and describe the dynamic mechanisms of gene regulation. In fact, it has been shown that the topology of gene regulations in yeast can dramatically change its structure during a cellular process (5). The dynamics of gene regulatory machinery have also been observed in nuclear microenvironments (6). On the other hand, approaches that can describe the dynamic behaviours of a process require time-course data, which are not available for many bio-medical problems such as cancer or diabetes. Indeed, disease samples (tissues or body fluids) are normally acquired for clinical purposes, such as diagnosis or treatment, rather than for research needs. Furthermore, a disease may span a period of months or years, thus making it infeasible to sample the entire disease process. Consequently, most gene-profiling data for medical problems are sample based, thereby impeding the application of dynamic approaches.

Although the elapsed time between disease onset and the collection of disease samples may be unknown, the samples are normally classified with staging information (e.g. cancer stages) that shows the clinical or pathological status of disease progression. In this article, we show that this staging information can be used to reproduce the gene-evolving trend and based on which to reconstruct dynamic GRN from the sample-based data by adopting two biologically plausible assumptions: the intra-stage steady-rate (or linear-dynamic) assumption and the continuity assumption, as illustrated in Figure 2. The intra-stage steady-rate assumption assumes that gene expression can be dynamic, and the dynamic profile should be associated with a linear trend within each stage of a process. The continuity assumption states that there are no discrete or abrupt changes in the gene profile even at the time of stage transition. The continuity assumption is natural because gene expression is an accumulated process, and thus cannot vary abruptly. Based on these two assumptions, we develop a dynamic cascaded method (DCM) to reconstruct the dynamic GRN from widely available sample-based transcriptional data.

Our DCM was first implemented on an *in silico* network as a simulation study. The performance of the DCM was confirmed by comparing it with static and dynamic approaches. The method was further applied to reconstruct the gene networks of hepatocellular carcinoma (HCC) progression. HCC is one of the most common cancers and causes of cancer deaths worldwide (7,8). The development of HCC is a complex multistep process involving several molecular and cellular changes. The precise mechanisms for these alterations are poorly understood (9,10). The DCM overcomes the limitations of current approaches and provides a new way of investigating the dynamic mechanisms of HCC progression using sample-based high-throughput data. The derived HCC networks were verified by functional analysis and network enrichment analysis. In addition, the modularity and network rewiring shown in the networks clearly characterize the dynamics of gene regulation during HCC progression.

## MATERIALS AND METHODS

Letting 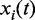 be the expression level of gene 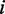 at time *t*, then the ordinary differential equation (ODE) of transcriptional kinetics can be written as (2)
(1)
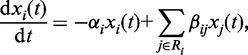

where 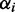 is the mRNA turnover rate (i.e. the probability that mRNA will be degraded in a given time interval), 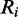 is the set of regulators of gene 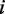 and 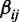 is the regulatory strength from gene 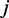 to gene 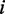. Equation (1) can be utilized in dynamic approaches to reconstruct dynamic GRNs based on time-course data, in which the model coefficients (degradation rate and regulatory strengths) can be determined by a linear regression method, such as LASSO (11,12).

If temporal information is not available, static approaches are virtually the only way of re-engineering gene networks. Static approaches assume that genes are expressed in steady state, so there is no evolving trend among the samples, i.e. 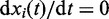. Therefore, (1) becomes 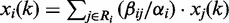, in which the model coefficients for the static GRN can be solved by linear regression after injecting the sample-based data.

**Figure 1.**
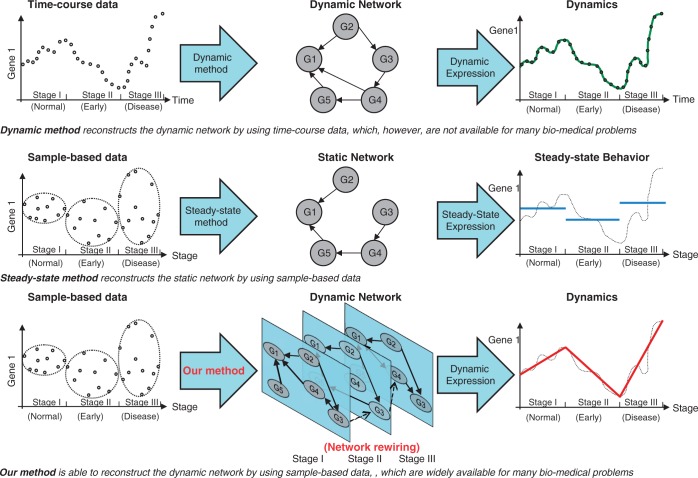
Schematic illustrations of network inference approaches. Dynamic approach (upper block) can reconstruct the dynamic network to describe the dynamic behaviours of transcriptional regulation by using time-course data, which is normally not available for many bio-medical problems; static approach (middle block) reconstruct static network which can only describe the (pooled average) static behaviours of transcriptional regulation by using sample-based data; our DCM (lower block) is able to reconstruct the dynamic network which describe the dynamic gene regulation even by using the sample-based data.

Instead of letting 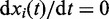 as in static approaches, our DCM assumes that the gene-evolving rate (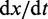) is a constant (but unknown) at each stage, corresponding to the intra-stage steady-rate assumption. By combining this with the continuity assumption, we can derive both the theoretical model and the algorithm for the DCM. The principles underlying the DCM, and the comparisons to dynamic and static approaches, are schematically illustrated in Figure 1.

Here, we briefly introduce the main model equations for the DCM. The details of this method can be found in Section 1 of the Supplementary Data. Let 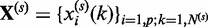 be the sample-based transcriptional data for stage *s* of a process, in which *P* is the number of genes, and 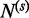 the number of samples. According to the intra-stage steady-rate assumption and the continuity assumption, we can construct an equation that connects the gene profiles of two consecutive stages:
(2)
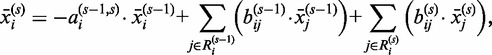

in which 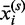 and 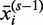 are the mean expressions of gene 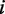 in stage *s* and *s*−1, respectively. The coefficients in (2) are related to the kinetic parameters in (1) with the following functions:
(3)
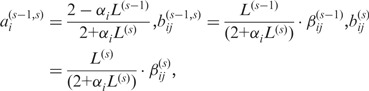

where 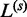 is the time span of stage *s*, 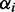 is the degradation rate and 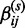 is the regulatory strength. Note that the inter-stage influence coefficient 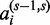 is a constant with a value between −1 and 1, as the degradation rate 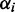 and the time span are both positive values. Equation (2) describes the pooled-average relationship of the gene expression status across stages, or it describes the average inter-stage dynamics of the GRN (i.e. average inter-stage dynamical GRN).

To describe the dynamic patterns within a stage, we define the 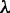-fraction of a stage as the proportional interpolation between the earliest and the latest time points of the stage (according to the intra-stage steady-rate assumption). If we use 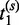 and 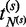 to denote the earliest and the latest time points of stage *s*, then the time of the 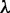-fraction in stage *s* can be expressed as 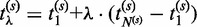. Following a series of transformations, we obtain the dynamic model equation (details of the mathematical manipulations can be found in Section 1 of the Supplementary Methods):
(4)
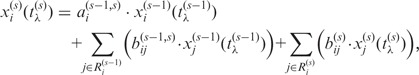

in which the gene expressions at the same (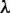) fraction of two consecutive stages are connected by a linear equation. Clearly, Equation (4) describes the inter-stage dynamics of the GRN (i.e. inter-stage dynamical GRN). Note that, during each stage, the intra-stage dynamics of the GRN (i.e. intra-stage dynamical GRN) is described by Equation (1).

**Figure 2.**
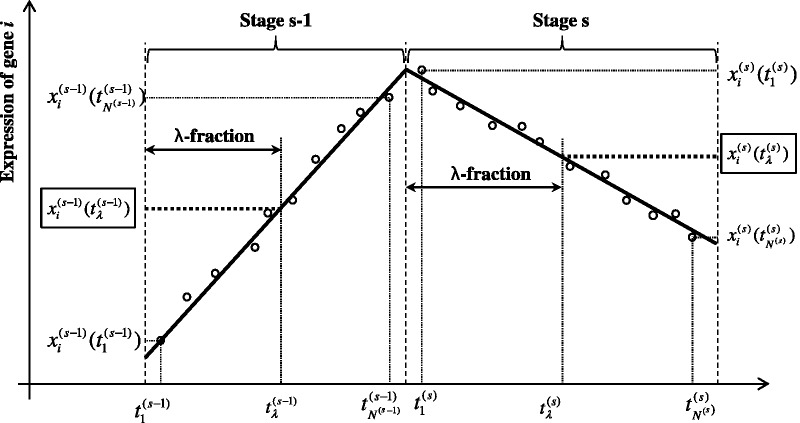
Schematic illustration of the dynamic cascaded model derived from the intra-stage steady-rate (or linear-dynamic) assumption and the continuity assumption. In the dynamic cascaded model defined in Equation (4), the time of *λ*-fraction of a stage is defined as the proportional interpolation between the earliest and the latest of the stage. The model variables, 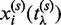 and 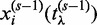, are highlighted with rectangles.

Theoretically, all of the coefficients 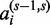, 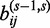, 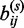 in (4) can be solved using a linear regression approach. Therefore, the existence and strength of the regulatory relationships (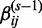
and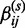) can be obtained according to the functions in (3). However, due to the lack of temporal information in the sample-based data, the model variable 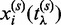 in (4) is not directly available. From Figure 2, we can see that 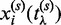 can be estimated by a quantile function of the samples from stage *s*, according to the linear-dynamic (steady-rate) behaviour of gene expression. In summary, if the gene evolves in an ascending trend (such as stage *s* − 1 in Figure 2), the quantile function will be 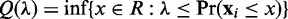; otherwise, if it evolves in a descending trend (such as stage *s*), the quantile function becomes 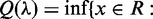

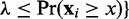
. Clearly, the specific assignment of the quantile function depends on the gene-evolving trend in each stage. To determine the gene-evolving trend, we introduce a method of gene-evolving trend analysis based on the intra-stage steady-rate assumption and the continuity assumption. Details of the gene-evolving trend analysis can be found in Section 2 of the Supplementary Methods.

During the process of network reconstruction, a bootstrapping strategy is employed to better utilize the limited samples. For each gene, the model equations in (4) with different settings of the fraction factor are produced from each bootstrap group, after which the model coefficients are determined using LASSO on all of the model equations.

In summary, the algorithm for the DCM is described as follows:

*Step 1*: Preprocess the original data to obtain the stage-wise sample-based transcriptional data 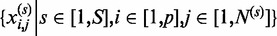.
*Step 2*: Perform the gene-evolving trend analysis described in Section 2 of the Supplementary Methods to determine the gene-evolving trend (ascending or descending) of each gene for each stage.
*Step 3*: Conduct the bootstrapping procedure and then produce the model equations in (4) of the inter-stage dynamical GRN.
  - Obtain a random group of bootstrapping samples for each gene at each stage;
  - Produce the model equations using different settings for the fraction factor (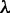) for each bootstrap group; and
  - Iterate (i) and (ii) to obtain the model equations.
*Step 4*: Estimate the model’s coefficients of (1), i.e., reconstruct the intra-stage dynamical GRN:
  - Based on the model equations obtained in Step 3, perform a LASSO regression to solve the model coefficients with different network sparsities;
  - Determine the model coefficients that have the most appropriate sparsity for different stages using a cross-validation approach; and
  - Reconstruct the GRNs described for each stage *s* by (1) according to 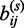, which is proportional to 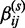.
*Step 5*: (Optional) repeat Steps 3 and 4 to calculate the confidence of the network connection.

## RESULTS

### Simulation study

Four *in silico* networks were constructed to simulate the regulatory behaviours of six genes during the four consecutive stages of a continuous process, in which the ODE, 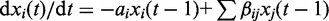, was used to represent the regulatory relationship. The topological structure of the four networks can be found in Supplementary Figure S1. Some common regulatory motifs (13) were randomly assigned to different networks, such as loop structure (3-5-4 in stage I; 2-6-3 and 1-4-5 and 2-6-3 in stage II; 2-6-4-3 in stage III; and 2-3-4 and 1-2-3-5-6 in stage IV), feed-forward structure (3-5-2 and 3-2-1-6 in stage I; 2-6-1 and 5-4-3 in stage III; and 3-5-4 in stage IV), and central structure (3-2-5-6 in stage I). Meanwhile, the degradation rates of all genes were set to 0.05, and the regulatory strengths were randomly selected to be either 0.1 or −0.1. The initial expression levels of all genes were set to 1.0 at the beginning of the process. The time spans of the four stages were set to 20, 30, 20 and 30, respectively. The continuity of the gene profile was ensured across the whole process, including the stage transition points. Using the above configurations, we generated the time-course gene profiles shown in Supplementary Figure S2. We then produced the stage-wise sample-based data by ignoring the time sequence of the samples while keeping their staging information.

The DCM algorithm was implemented to reconstruct the GRN by the produced sample-based data so as to evaluate the effectiveness of our method. First, the gene trend analysis was performed to reproduce the gene-evolving trend (ascending or descending) of each gene in each stage. Then, the bootstrapping approach was performed to generate bootstrap groups. For each group, model equations in (4) were produced, corresponding to the gene-evolving trend determined in the first step and the settings of the fraction factor. Multiple fraction factors were used to ensure the linearity of the model equations at different check-points. In the absence of theoretical proof, we heuristically set the fraction factors to be 0, 0.5 and 1.0. The fraction factor of 0.5 corresponded to the middle of a stage, whereas the settings 0 and 1.0 were responsible for the linearity at the beginning and end of a stage. In addition to the interpolation of a stage, we generally found it useful to control the linearity of the model equations on the extrapolation of a stage. Two linear extrapolations, including the head-end extrapolation 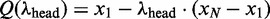 and the tail-end extrapolation 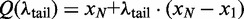 were used. In general, 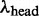 and 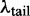 should be large enough to control the linearity at the far ends of a stage. Here, both 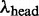 and 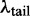 were set to 4.0 (the DCM performed almost equally in simulations when these two parameters were set within the range 3.0–8.0). Finally, a total of 200 bootstrap groups were generated and 1000 corresponding model equations were obtained. The model coefficients 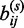 were then solved by LASSO regression, in which the model parameter 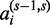 was set to a value between −1 and 1. Based on the beta matrix obtained by LASSO, cross validation was implemented to determine the relevant sparsity of the network topology. Supplementary Figure S3 shows a group of GRNs obtained from an iteration of the above process.

The performance of the network reconstruction is normally measured by the receiver operating characteristic (ROC) curve. The ROC shows the true positive rate (sensitivity) as a function of the false positive rate (1 − specificity) corresponding to different cut-offs of the decision threshold (14). Based on the ROC curve, the area under the curve (AUC) can be calculated to represent the predictive ability of a network inference approach. In addition, the *Z*-score, which describes the difference between a prediction and a random guess, and the *P*-value of the significance test, were used to indicate the significance of a network inference. The *F*_2_-score and Matthews Correlation Coefficient (MCC, a balanced measure of performance) (15) were computed as a reference indicator, with higher values indicating better performance for both measures.

In the simulation study, the modelling process was repeated 100 times to cancel the randomness effect. The overall AUC was then calculated on the networks for all four stages. All of the above analyses were performed programmatically in MATLAB 7.12. The predictive performance of the DCM is presented in Table 1, which shows that the overall AUC was 0.75 (the performance obtained with non-optimal settings), indicating the good predictive ability of the DCM. The *P-*value of the *Z*-test was 0.0065, suggesting that the network inference was significantly different from random guesses. The overall MCC value was also much >0, further confirming that the network prediction was significant.

**Table 1.** Performance metrics of the DCM on the sample-based data of the simulation study

	Stage I	Stage II	Stage III	Stage IV	Overall
					
AUC	0.72	0.82	0.68	0.73	0.75 ± 0.056
*P*-value	<0.0001	<0.0001	0.0029	<0.0001	0.0065
*F*_2_-score	0.74 ± 0.000	0.81 ± 0.020	0.67 ± 0.041	0.77 ± 0.038	0.75 ± 0.058
MCC	0.44 ± 0.017	0.60 ± 0.035	0.39 ± 0.033	0.38 ± 0.030	0.46 ± 0.092

**Figure 3.**
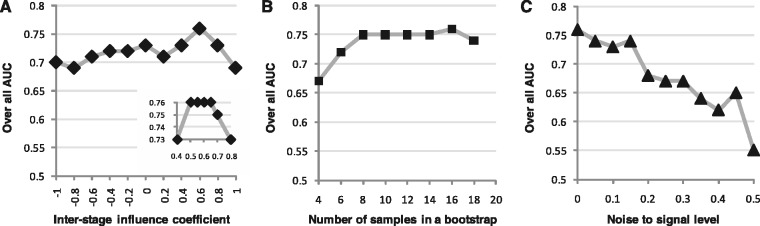
Results of the performance test on *in silico* network of the simulation study. (**A**) Result of sensitivity test on different settings of the inter-stage influence coefficient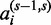. The overall AUCs were generally >0.7 for most of settings. In particular, the overall AUC reached the peak value of 0.76 when 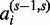 was set to a value between 0.5 and 0.65. (**B**) Result of sensitivity test on different settings for number of samples in a bootstrap group. The overall AUCs were above 0.75 when the number of samples in a bootstrapping group was set in the range from 8 to 18 (recall that the number of samples for stage I, II, III and IV are 20, 30, 20 and 30, respectively). (**C**) Result of robustness test on different levels of noise corruption to the transcriptional data. The overall AUCs were over 0.73 when the noise-to-signal level was smaller than 0.15. When the noise level increased to 0.2–0.3, the overall AUC gradually dropped to 0.67. It further dropped to 0.62 when the noise level increased to 0.4, and eventually became unacceptable (∼0.55) when the noise level reached 0.5.

We have conducted the performance test on sensitivity and robustness of the DCM. Figure 3a shows the result of sensitivity test in corresponding to different settings for the inter-stage influence coefficient 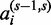, in which most of the overall AUCs were over 0.7. In particular, the overall AUC remained on the peak value of 0.76 when 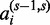 was set to a value in the range from 0.5 to 0.65.

The result of sensitivity test on various settings for the number of samples in a bootstrap group is shown in Figure 3B, in which it can be found that the DCM is not sensitive to this parameter. The overall AUCs were above 0.75 when the number of samples in a bootstrap group was set in the range from 8 to 18 (recall that the number of samples for stage I, II, III and IV are 20, 30, 20 and 30, respectively).

The robustness of the DCM was tested by corrupting the transcriptional data with different levels of random noise. In this test the number of samples in each bootstrap group was set to 16, and 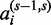 was fixed at 0.6. The trend of overall AUCs versus different levels of noise corruption is shown in Figure 3C. It can be seen that the performance of DCM was quite robust (overall AUCs were over 0.73) when the noise-to-signal level was smaller than 0.15. When the noise level increased to 0.2–0.3, the overall AUC gradually dropped to 0.67. It further dropped to 0.62 when the noise-to-signal level increased to 0.4, and eventually became unacceptable (∼0.55) when the noise level reached 0.5.

As a comparison, we implemented the static (steady-state) approach using the sample-based transcriptional data to reconstruct the GRNs. The model equations were obtained by letting 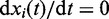 in (1), and were then solved by LASSO. Cross validation was used to determine the appropriate sparsity of the network topology. The performance of the static approach is presented in Table 2. The overall AUC was 0.56 and the *P*-value of the significance test was 0.28, suggesting that the predictive ability was quite poor. This result implies that the task of network reconstruction using sample-based data is quite difficult. Our DCM, however, significantly improved the predictive ability of network inference from 0.56 to above 0.75 (achieved with fair settings) with a net increase of around 34%.

**Table 2.** Performance metrics of stage-wise static (steady-state) method on the sample-based data of the simulation study

	Stage I	Stage II	Stage III	Stage IV	Overall
					
AUC	0.57 ± 0.033	0.56 ± 0.052	0.51 ± 0.031	0.61 ± 0.016	0.56 ± 0.056
*P*-value	0.034	0.249	0.747	<0.0001	0.284
*F*_2_-score	0.66 ± 0.002	0.72 ± 0.018	0.68 ± 0.019	0.67 ± 0.019	0.68 ± 0.027
MCC	0.34 ± 0.034	0.36 ± 0.035	0.44 ± 0.000	0.37 ± 0.018	0.37 ± 0.055

We also reconstructed the dynamic GRN based on time-course data using ODEs (16). Again, the parameters in the model equations were solved by LASSO and then determined by cross validation. The performance of the dynamic approach is presented in Table 3. The overall AUC of the dynamic approach was around 0.94, suggesting a performance improvement of 68% (i.e. 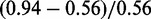) compared with the steady-state approach, due to the use of the temporal information. In other word, our DCM can recover around 50% of the (ignored or missed) dynamic patterns, i.e. 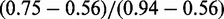. This result suggests that the DCM can appropriately exploit the staging information to reproduce a significant portion of the temporal information for reconstructing the dynamic GRN, by modelling the linear-dynamic behaviour of gene regulation based on the intra-stage steady-rate assumption and the continuity assumption.

**Table 3.** Performance metrics of dynamic method on the time-course data of the simulation study

	Stage I	Stage II	Stage III	Stage IV	Overall
					
AUC	0.97 ± 0.000	0.98 ± 0.000	0.88 ± 0.000	0.93 ± 0.000	0.94 ± 0.040
*P*-value	<0.0001	<0.0001	<0.0001	<0.0001	<0.0001
*F*_2_-score	0.98 ± 0.000	0.97 ± 0.000	0.85 ± 0.000	0.91 ± 0.000	0.93 ± 0.052
MCC	0.93 ± 0.000	0.92 ± 0.000	0.78 ± 0.000	0.75 ± 0.000	0.85 ± 0.082

### Application of GRN modelling to HCC progression

Sample-based gene-profiling data of HCC progression were extracted from (17), in which 10 normal tissue samples were obtained from healthy livers and 65 disease samples were obtained from 38 patients with HCV infection, representing the stepwise carcinogenic process from pre-neoplastic lesions to HCC. The disease samples were categorized into 5 consecutive stages along the carcinogenic process: 13 samples for the cirrhotic stage, 17 for the dysplastic stage, 18 for early HCCs and 17 for advanced HCCs.

Among the signalling pathways that may be influenced by HCC, we were particularly interested in the cell cycle pathway because it is the most affected pathway in HCC and has the highest correlation with cancer progression (17). From the cell cycle signalling pathway, we extracted 52 TFs that showed significant changes (*P* < 0.05) during the HCC process to be the network nodes.

Before DCM modelling, we conducted a gene-evolving trend analysis for each gene. Supplementary Figure S5 shows the partial results of the gene-evolving trends for some hub genes (hub genes can be identified according to the size of the nodes in Figure 6, as illustrated below). Supplementary Figure S5 confirmed that the continuity assumption generally held.

**Figure 4.**
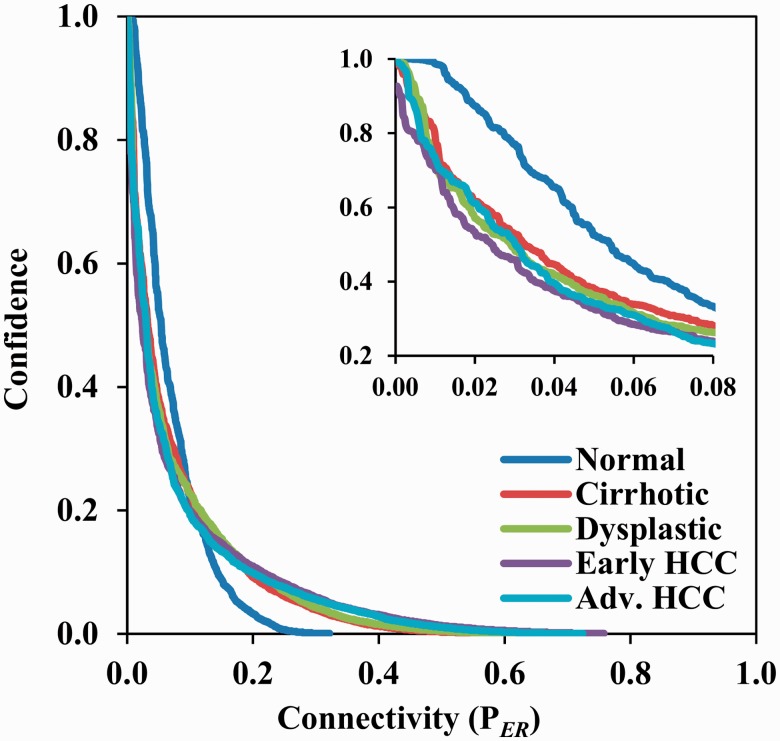
The curves of the connection confidence versus the network connectivity for all five stages during HCC progression. Of the 2652 possible (without self) connections for a set of 52 genes in the cell cycle signalling pathway, most connections have very low certainty of occurrence. The connection confidence decreased exponentially as the network connectivity increased from 0 to 1.0, also the curves of the four disease stages (cirrhotic, dysplastic, early HCC and the advanced HCC) have similar shapes, which are apparently different from the one of the normal stage.

During the bootstrapping process, seven samples (i.e. roughly half of the samples for each stage) were randomly extracted from each stage to form bootstrap groups. No particular efforts were made to determine the settings of the fraction factor; we simply used the same settings that we used in the simulation study, i.e., 0, 0.5 and 1.0 for interpolation, and 4.0 for both head-end and tail-end extrapolation. We conducted 5 bootstraps and thus obtained 25 model equations for each gene. Consequently, 1300 model equations were generated for 52 genes (i.e. 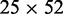). The model coefficients were solved by LASSO regression. A network was constructed using the top 185 connections [we reserved 185 connections to ensure that all 52 nodes had an opportunity to be included in the derived network, according to the Erdős–Rényi graph theory (18)]. The process was repeated 1000 times, and correspondingly 1000 networks were obtained. The confidence of a connection was calculated as its occurring frequency in all the 1000 networks. The foregoing analyses were performed in Matlab running in parallel on 20 server computers (HP Proliant DL360 G7, Dual 6-core Intel Xeon X5650 with a CPU frequency of 2.66 GHz, 12 MB L3 Cache and 32 G memory) for around 20 h. Figure 4 shows the curves of the connection confidence versus the network connectivity for all five stages. The network connectivity was defined as the number of connections reserved in a network. Implementing a threshold either on the connection confidence or the network connectivity allowed us to restore the connections of a gene network. Of the 2652 possible (without self) connections for a set of 52 genes in the cell cycle signalling pathway, most connections have very low confidence. For example, at confidence threshold of 0.5 there are 143, 86, 76, 64 and 79 connections in the networks of normal, cirrhotic, dysplastic, early HCC and the advanced HCC stage. Correspondingly the network connectivity on these 5 stages are 0.054, 0.032, 0.029, 0.024 and 0.030, respectively. Moreover, the connection confidence decreases exponentially as the network connectivity increases from 0 to 1.0, and the curves of the four disease stages (cirrhotic, dysplastic, early HCC and the advanced HCC) have similar shapes, which are apparently different from the one of the normal stage.

#### Enrichment analysis

**Figure 5.**
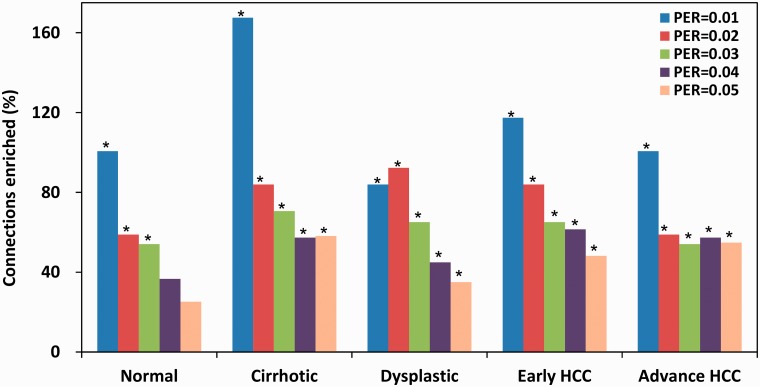
Network enrichments on the known molecular interactions during HCC progression. Significant enrichments were marked with asterisks. Network enrichments are significant for all five stages at network connectivity of 0.01 (26 connections), 0.02 (53 connections) and 0.03 (79 connections). Network enrichments remained to be significant for the four HCV-infected networks (cirrhotic, dysplastic, early HCC and advanced HCC) at network connectivity of 0.04 and 0.05. The highest enrichment was accomplished with a network connectivity of 0.01.

The derived networks can be verified by enrichment analysis. The known molecular interactions among the 53 TFs were extracted from three databases: the PINA (protein interaction network analysis) (19), KEGG (Kyoto encyclopaedia of genes and genomes) (20) and ITFP databases (21), in which there were 342, 274 and 144 connections, respectively. Consequently, 610 distinct interactions were retained after removing the overlaps. Network enrichment was defined as the ratio of the proportion of known interactions over the baseline proportion of a random guess (in this case, the baseline was 0.23, i.e. 610 known interactions among 2652 connections). Network enrichment is considered to be significant if the *P*-value of the significance test is smaller than 0.05. Figure 5 shows the network enrichments at different levels of network connectivity (0.01, 0.02, 0.03, 0.04 and 0.05), in which the significant enrichments are marked with asterisks. Figure 5 shows that the network enrichments were significant for all five stages when network connectivity was 0.01 (i.e. 26 connections derived from 2652 connections), 0.02 (53 connections) or 0.03 (79 connections). Meanwhile, network enrichments in the four HCV-infected networks (cirrhotic, dysplastic, early HCC and advanced HCC) remained significant when the network connectivity level was 0.04 and 0.05. The highest network enrichment was accomplished at connectivity level of 0.01, in which around half of the network connections were known interactions. More detailed results of the network enrichment analysis are presented in Supplementary Table S1 in the Supplementary Data.

#### Functional analysis

**Figure 6.**
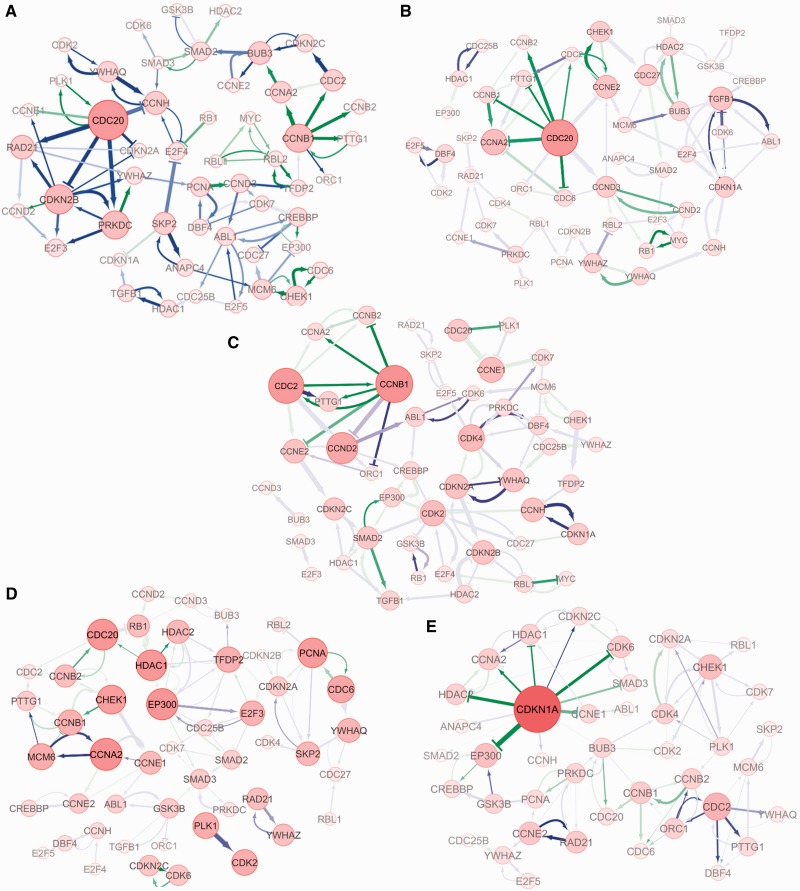
The GRNs reconstructed by our DCM using the sample-based transcriptional data of the five consecutive stages during HCC progression. (**A**) The reconstructed GRN for the normal stage; (**B**) The reconstructed GRN for cirrhotic stage of HCC; (**C**) The reconstructed GRN for the dysplastic stage of HCC; (**D**) The reconstructed GRN for the early HCC stage; and (**E**) The reconstructed GRN for the advanced HCC stage.

Five DCM networks were reconstructed with the connectivity level of 0.03 (79 connections reserved in each network) to describe the dynamical regulations during HCC progression. The networks were visualized with Cytoscape (http://www.cytoscape.org) and are shown in Figures 6A–E. In these figures, the size of each node is drawn in proportion to the sum of the products of the confidence and strength over all the direct incoming and outgoing connections, representing the activity of a gene in a network. The activation and inhibition are denoted with an arrow (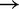) and stop (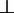), respectively. The level of the grey colour of an edge is in proportion to its confidence level, and the line width is associated with the strength of the regulation. All known interactions are drawn in green, and novel ones are in blue.

As expected, in the network of the normal stage most of the hub genes and key modules are related to the regular activities of cell cycle regulation. For example, the hub gene CDC20 encodes the cell-division cycle protein 20, which is an essential regulator of cell division in humans (22,23). As indicated in the network, CDC20 also promotes cell cycle progression by suppressing CDKN2B (the blue edge represents a novel connection), which encodes a cell growth inhibitor. Another active module of CCNB1 and CDC2 regulates the activity of mitosis through the Cyclin B1-Cdc2 kinase (24).

In the cirrhotic network, in addition to the CDC20 module that regulates the regular cell cycle activities, a new hub gene, TGFB1, became active. It has been reported that TGF-beta1 (encoded by TGFB1) is significantly upregulated by the HCV core protein (25), and the upregulation of TGFB1 can inhibit the secretin and activity of many cytokines and various interleukins, and decrease the expression levels of cytokine receptors to down-regulate the activity of immune cells (26,27). Moreover, the novel regulation from ABL1 to TGFB1 in the network can be explained by Cayne and Bergelson (28), in which it was reported that virus-induced Abl kinase allows the coxsackievirus to enter through epithelial tight junctions.

In the dysplastic network, the regular cell cycle regulatory modules CCNB1 (which encodes Cyclin B1) and CDC2 (which encodes Cdk1) remain active, and the new modules CDK2 and CDK4 also become active at this stage. It has been reported that the activation of CDK2 and CDK4 can only occur in cells expressing full-length HCV (HCR6-Rz) RNA after 44 days (29). Indeed, the just-in-time appearance of CDK2 and CDK4 in the dysplastic network suggests that the DCM can capture the dynamic gene regulation patterns in HCC progression. Moreover, the activated pathways of CDKN1A-CDK2-E2F4-RBL1 and CDKN2A-CDK2-E2F5 found in the network confirm the findings in (30), which reported that the CKI-CDK-E2F-Rb pathway (note that CDKN1A and CDKN2A are both CKIs—Cyclin dependent kinase inhibitor genes, and RBL1-encoded protein is similar in sequence and function to the product of the RB1 gene) is activated by HCV infection. Other links, such as regulation from SMAD2 to TGFB1 (31), regulation from HDAC1 to SMAD2 (32) and bidirectional regulations between SMAD2 and EP300 (32), have also been confirmed.

The most prominent difference between early HCC and the dysplastic network is the module comprising EP300, HDAC1, HDAC2, TFDP2 and E2F3. It has been recently reported that HDAC inhibitors and EP300 (which encodes the E1A binding protein) are directly responsible for the up-regulation of microRNA-24 (miR-224), which is one of the most commonly up-regulated microRNAs in HCC that affect crucial cellular processes such as apoptosis and cell proliferation (33). Meanwhile, the up-regulation of TDDP2 (which encodes the transcription factor DP2) and E2F3 in HCC have also been confirmed (34). Furthermore, most of the connections in the PCNA, CDC6 and CDKN2A module are known molecular interactions. It has been reported that PCNA express different isoforms in human HCCs compared to those in cirrhosis, suggesting that PCNA may play a role in HCC genesis (35). The cell cycle modules are still one of the most active modules, including the genes of CCNA2, MCM6, CHEK1 and CCNB. CCNA2 and MCM6 are highly conserved genes, responsible for regular cell cycle regulation. Chk1 (encoded by CHEK1) is a kinase that phosphorylates cdc25, and the phosphatase cdc25 can dephosphorylate cdk1, which activates Cyclin B1 (CCNB1).

In the advanced HCC network, the main function of the cell cycle signalling pathway becomes tumour suppressing. The dominant gene is CDKN1A, by which the induced protein p21 is a potent Cyclin-dependent kinase (cdk) inhibitor. The expression of CKDN1A is tightly controlled by the tumour suppressor protein p53, through which p21 mediates the p53-dependent cell cycle G_1_ phase arrest in response to a variety of stress stimuli (36,37). Like p53, p21 is a tumour suppressor. In the network, CDKN1A/p21 inhibits EP300, HDAC1 and HDAC2, and thereby down-regulates the expression of microRNA-24 (one of the most commonly up-regulated microRNAs in HCC) (33). Meanwhile, CDKN1A/p21 also inhibits CCNE and CCNA (both up-regulated in HCC) (17). Furthermore, the PCNA, PRKDC and RAD21 module is important to DNA repair as it also helps to suppress tumours. For example, PCNA-induced protein is ubiquitinated in response to DNA damage, and is involved in the RAD6-dependent DNA repair pathway (38). DNA-PKcs induced by PRKDC is required for the non-homologous end-joining (NHEJ) pathway of DNA repair, which rejoins double-strand breaks. The double-strand-break repair protein rad21 homologue is a protein that is encoded by the RAD21 gene in humans (39).

## DISCUSSION

Conventional dynamic network inference approaches are not applicable to sample-based data, which is the most common type of data for many bio-medical processes such as human diseases (40,41). To overcome the limitations of dynamic approaches, we developed a DCM to reconstruct dynamic gene networks from sample-based data. A simulation study showed that our method was more accurate in recovering the true interactions compared with the existing approach using the same data.

Our method was applied to modelling the HCC progression using the existing sample-based transcriptional data. The resulting networks were verified by network enrichment analysis and functional analysis. These networks significantly and robustly enriched the known interactions at different levels of network connectivity. In general, the known interactions were more condensed in sparser networks, suggesting that the confidence measure can accurately reflect the true regulatory relationship. In particular, when the network was very sparse with a network connectivity level of 0.01 (i.e. with only 26 connections preserved), around half of the predicted connections can be associated with known molecular interactions.

Modularity and network rewiring phenomena were observed in the DCM networks. The activation and inhibition of different hub genes and modules reflect the dynamic alterations of network functionality that may be responsible for, or responsive to HCC progression. For instance, the TGFB1 module was turned on by the immune system when triggered by HCV infection, as shown in Figure 6B, which coincides well with the characteristic of the cirrhotic stage. Activations of CDK2 and CDK4, which are transient patterns that only occur after a certain period successive to HCV infection, were clearly identified in the dysplastic network, as shown in Figure 6C. Transient patterns were also observed in the early HCC network, including activation of the HDAC and EP300 module and the PCNA module, suggesting that the tissue was evolving towards HCC. Moreover, the dominant role of CDKN1A in the advanced HCC network suggests that the main function of the cell cycle signalling pathway, have evolved from cell cycle regulating to tumour suppressing.

Another thing that we observed is that the DCM networks may retain significantly different levels of network connectivity for different stages of HCC, even with the same confidence threshold. For instance, with a confidence threshold of 0.5, there were 143, 86, 76, 64 and 79 connections reserved for the normal, cirrhotic, dysplastic, early HCC and advanced HCC stages. The normal stage retained the largest number of connections, suggesting that gene regulation in the normal stage was more stable (or in other word, gene-evolving speed is slower) than in the disease stages. Similarly, the early HCC network retained the fewest connections, suggesting that the speed of gene evolution in early HCC may be faster than in other stages.

From the viewpoint of dynamic theory, disease progression can be considered a state transition process (40), from a normal state, to a disease onset state and its advanced state. In the case of HCC, the stable trajectory of gene regulation in the normal system, once disturbed by tumour initiation, may gradually drifted to a dynamic trajectory of cancer development towards the terminal HCC stage. The overall trajectory of gene transcription may be highly nonlinear and time dependent, hence time-course measurement is naturally an ideal way of describing this dynamic behaviour. To that end, the DCM model can be regarded as an alternative to the time-course approaches, by using a stage-cascaded linear approximation to estimate the actual nonlinear trajectory.

Our method provides a new way of re-engineering dynamic networks even when time-course data are not available. We expect that it will bring maximal benefits in studying gene regulations during the dynamic processes of many chronic genetic diseases, such as cancer, diabetes, etc. However, the improvement of the DCM over the steady-state approach may be minor when the state spaces are truly or nearly steady. In addition, the performance of the DCM may deteriorate if the samples are unevenly distributed in a stage, e.g. when the samples are concentrated within a narrow range of a stage. The programs and relevant data are available from http://www.comp.hkbu.edu.hk/∼hlzhu/NAR_codes.html.

## SUPPLEMENTARY DATA

Supplementary Data are available at NAR Online: Supplementary Table 1, Supplementary Figures 1–5 and Supplementary Methods.

## FUNDING

Research Grants Council of Hong Kong [212111]; Startup Grant of Science Faculty of Hong Kong Baptist University [3840030]; NSFC [91029301, 61134013, 61072149 (in part)]; Chief Scientist Program of SIBS of CAS [2009CSP002 (in part)]; FIRST program from JSPS initiated by CSTP (in part). Funding for open access charge: Startup Grant of Science Faculty of Hong Kong Baptist University [3840030].

*Conflict of interest statement*. None declared.

## Supplementary Material

Supplementary Data
