# Supplementary material for: Reconstructing dynamic gene regulatory networks from sample-based transcriptional data
Source: Nucleic Acids Res. 2012 Sep 21;40(21):10657–67. doi: 10.1093/nar/gks860 (PMC3510506; doi:10.1093/nar/gks860)
Supplement: Supplementary Data [file supp_40_21_10657__index.html]

Reconstructing dynamic gene regulatory networks from sample-based transcriptional data — Reconstructing dynamic gene regulatory networks from sample-based transcriptional data — Supplementary Data 

# Reconstructing dynamic gene regulatory networks from sample-based transcriptional data

## Supplementary Data

files

**Files in this Data Supplement:**

- Supplementary Data - pdf file
