# Supplementary material for: Reconstructing dynamic gene regulatory networks from sample-based transcriptional data
Source: Nucleic Acids Res. 2012 Sep 21;40(21):10657–67. doi: 10.1093/nar/gks860 (PMC3510506; doi:10.1093/nar/gks860)
Supplement: Supplementary Data [file supp_gks860_nar-01466-n-2012-File008.pdf]

Reconstructing dynamic gene regulatory networks from sample-based transcriptional data

Hailong Zhu[1,*], R. Shyama Prasad Rao[1], Tao Zeng[2], Luonan Chen[2,*]

[1] Department of Computer Science, Hong Kong Baptist University, Kowloon Tong, Hong Kong
[2] Key Laboratory of Systems Biology, SIBS-Novo Nordisk Translational Research Centre for PreDiabetes, Shanghai Institutes of Biological Sciences, Chinese Academy of Sciences, China

* To whom correspondence should be addressed:
H Zhu: Tel: (852) 3411 7636, Fax: (852) 3411 7892, Email: hlzhu@comp.hkbu.edu.hk
L Chen: Tel: (86) 21-6436 5937, Fax: (86) 21-5492-0120, Email: lnchen@sibs.ac.cn

E-mail:
rsprao@comp.hkbu.edu.hk (RSP Rao)
zt_2003@163.com (T Zeng)

Supplementary Methods

Section one: Theory and method of model construction

Letting $x_i^{(s)}(t)$ be the transcriptional level of gene i at time *t*, the ordinary differential equation (ODE) of transcriptional kinetics can be written as [2]:

$$r_i^{(s)}(t) = \frac{dx_i^{(s)}(t)}{dt} = -\alpha_i^{(s)} x_i^{(s)}(t) + \sum_{j \in R_i^{(s)}} \beta_{ij}^{(s)} x_j^{(s)}(t) \tag{1}$$

where $r_i^{(s)}(t)$ is the evolving rate of the expression of gene i at time t, α_i is the mRNA turnover rate (i.e. i.e. the probability that mRNA will be degraded in a given time interval), R_i is the set of regulators of the gene i, and β_{ij} is the regulatory strength from gene j to gene i. Equation (1) is generally used to describe a dynamical gene regulatory network (GRN). (1) can be rewritten as a difference equation:

$$r_i^{(s)}(t_k) = \frac{x_i^{(s)}(t_{k+1}) - x_i^{(s)}(t_k)}{\Delta t_k} = -\alpha_i^{(s)} x_i^{(s)}(t_k) + \sum_{j \in R_i^{(s)}} \beta_{ij}^{(s)} x_j^{(s)}(t_k) \tag{2}$$

in which $\Delta t_k = t_{k+1} - t_k$. Equation (2) can be employed in a dynamical approach to reconstruct the dynamic network based on the time-course data.

Suppose there are $N^{(s)}$ samples obtained at unknown times of $t_1, t_2, \cdots, t_k, \cdots t_{N^{(s)}}$ in stage *s*, and time span of which is denoted by $L^{(s)}$. If we assume that these samples are independently and identically distributed (*i.i.d.*) within a stage, then the average time interval between two neighbouring samples can be expressed as $\Delta t = L^{(s)} / (N^{(s)} - 1) + \varepsilon$, where ε is of a zero-mean normal distribution, i.e. $\varepsilon \sim N(0, \delta^2)$. Therefore, (2) can be converted to

$$x_i^{(s)}(t_{k+1}) - x_i^{(s)}(t_k) = r_i^{(s)}(t_k) \cdot L^{(s)} / (N^{(s)} - 1) + r_i^{(s)}(t_k) \cdot \varepsilon_k \tag{3}$$

After performing summation of all of the above equations in this stage, we get

$$x_i^{(s)}(t_{N^{(s)}}) - x_i^{(s)}(t_1) = \left(\sum_{k \in G^{(s)}} r_i^{(s)}(t_k) \right) \cdot \frac{L^{(s)}}{N^{(s)} - 1} + \sum_{k \in G^{(s)}} r_i^{(s)}(t_k) \cdot \varepsilon(t_k) \tag{4}$$

where t_1 and $t_{N^{(s)}}$ are the starting and ending time of stage *s*, and $G^{(s)} = [1, N^{(s)}]$ denotes the set of samples in this stage. Since the distribution of $r_i^{(s)}(t_k)$ has no relationship with ε, the second item of the right side of (4), $\sum \left(r_i^{(s)}(t_k) \cdot \varepsilon(t_k) \right)$, should have a zero mean. Therefore, we get:

$$E\left(x_i^{(s)}(t_{N^{(s)}}) - x_i^{(s)}(t_1) \right) = E\left(\left(\sum_{k \in G^{(s)}} r_i^{(s)}(t_k) \right) \cdot \frac{L^{(s)}}{N^{(s)} - 1} \right) \tag{5}$$

Substitutes (2) into (5), then we get:

$$\frac{\hat{x}_i^{(s)}(t_{N^{(s)}}) - \hat{x}_i^{(s)}(t_1)}{L^{(s)}} = \frac{1}{N^{(s)} - 1} \left(-\alpha_i^{(s)} \sum_{k=1}^{N^{(s)}-1} x_i^{(s)}(t_k) + \sum_{j \in R_i^{(s)}} \left(\beta_{ij}^{(s)} \cdot \sum_{k=1}^{N^{(s)}-1} x_j^{(s)}(t_k) \right) \right) \tag{6}$$

where $\hat{x}_i^{(s)}(t_{N^{(s)}})$ and $\hat{x}_i^{(s)}(t_1)$ are the means of $x_i^{(s)}(t_{N^{(s)}})$ and $x_i^{(s)}(t_1)$, respectively.

According the intra-stage steady-rate assumption, we have

$$c_i^{(s)} = \frac{\hat{x}_i^{(s)}(t_{N^{(s)}}) - \hat{x}_i^{(s)}(t_1)}{L^{(s)}} = 2 \cdot \frac{\hat{x}_i^{(s)}(t_{N^{(s)}}) - \overline{x}_i^{(s)}}{L^{(s)}} = 2 \cdot \frac{\overline{x}_i^{(s)} - \hat{x}_i^{(s)}(t_1)}{L^{(s)}} \tag{7}$$

$$c_i^{(s-1)} = \frac{\hat{x}_i^{(s-1)}(t_{N^{(s-1)}}) - \hat{x}_i^{(s-1)}(t_1)}{L^{(s-1)}} = 2 \cdot \frac{\hat{x}_i^{(s-1)}(t_{N^{(s-1)}}) - \overline{x}_i^{(s-1)}}{L^{(s-1)}} = 2 \cdot \frac{\overline{x}_i^{(s-1)} - \hat{x}_i^{(s-1)}(t_1)}{L^{(s-1)}} \tag{8}$$

Meanwhile, we have $\hat{x}_i^{(s-1)}(t_{N^{(s-1)}}) \cong \hat{x}_i^{(s)}(t_1)$ according to the continuity assumption. Thus (7) and (8) can be converted to:

$$2 \cdot \frac{\overline{x}_i^{(s)} - \hat{x}_i^{(s)}(t_1)}{L^{(s)}} = -\alpha_i^{(s)} \sum_{k \in G^{(s)}} x_i^{(s)}(t_k) \Big/ N^{(s)} + \sum_{j \in R_i^{(s)}} \left(\beta_{ij}^{(s)} \cdot \sum_{k \in G^{(s)}} x_j^{(s)}(t_k) \Big/ N^{(s)} \right), \text{ and} \tag{9}$$

$$2 \cdot \frac{\hat{x}_i^{(s-1)}(t_{N^{(s-1)}}) - \overline{x}_i^{(s-1)}}{L^{(s-1)}} = -\alpha_i^{(s-1)} \frac{1}{N^{(s-1)} - 1} \sum_{k=1}^{N^{(s-1)}-1} x_i^{(s-1)}(t_k) + \sum_{j \in R_i^{(s-1)}} \left(\frac{\beta_{ij}^{(s-1)}}{N^{(s-1)} - 1} \cdot \sum_{k=1}^{N^{(s-1)}-1} x_j^{(s-1)}(t_k) \right) \tag{10}$$

Because $\hat{x}_i^{(s-1)}(t_{N^{(s-1)}}) \cong \hat{x}_i^{(s)}(t_1)$, $\overline{x}_i^{(s)} = \sum_{k \in G^{(s)}} x_i^{(s)}(t_k) \Big/ N^{(s)}$, and $\overline{x}_i^{(s-1)} = \sum_{k \in G^{(s-1)}} x_i^{(s-1)}(t_k) \Big/ N^{(s-1)}$, we get:

$$\overline{x}_i^{(s)} = -\frac{\alpha_i^{(s-1)} L^{(s-1)} - 2}{\alpha_i^{(s)} L^{(s)} + 2} \cdot \overline{x}_i^{(s-1)} + \sum_{j \in R_i^{(s-1)}} \left(\frac{L^{(s-1)} \beta_{ij}^{(s-1)}}{\alpha_i^{(s)} L^{(s)} + 2} \cdot \overline{x}_j^{(s-1)} \right) + \sum_{j \in R_i^{(s)}} \left(\frac{L^{(s)} \beta_{ij}^{(s)}}{\alpha_i^{(s)} L^{(s)} + 2} \cdot \overline{x}_j^{(s)} \right) \tag{11}$$

By letting $a_i^{(s-1,s)} = \frac{\alpha_i^{(s-1)} L^{(s-1)} - 2}{\alpha_i^{(s)} L^{(s)} + 2}$, $b_{ij}^{(s-1,s)} = \frac{L^{(s-1)}}{(\alpha_i^{(s)} L^{(s)} + 2)} \cdot \beta_{ij}^{(s-1)}$, and $b_{ij}^{(s)} = \frac{L^{(s)}}{(\alpha_i^{(s)} L^{(s)} + 2)} \cdot \beta_{ij}^{(s)}$, we can obtain

$$\overline{x}_i^{(s)} = -a_i^{(s-1,s)} \cdot \overline{x}_i^{(s-1)} + \sum_{j \in R_i^{(s-1)}} \left(b_{ij}^{(s-1)} \cdot \overline{x}_j^{(s-1)} \right) + \sum_{j \in R_i^{(s)}} \left(b_{ij}^{(s)} \cdot \overline{x}_j^{(s)} \right) \tag{12}$$

where $a_i^{(s-1,s)}$ is the inter-stage influence coefficient, $b_{ij}^{(s-1,s)}$ is proportional to the regulatory strength $\beta_{ij}^{(s-1)}$, and $b_{ij}^{(s)}$ is proportional to $\beta_{ij}^{(s)}$. Cleary, equation (12) describes the average inter-stage dynamics of a GRN.

Since gene expression varies linearly in a stage, so there have $\overline{x}_i = (x_i(t_N) + x_i(t_1))/2$ and $\overline{x}_j = (x_j(t_N) + x_j(t_1))/2$ for each stage. Therefore, there have

$$\begin{aligned} (x_i^{(s)}(t_{N^{(s)}}^{(s)}) + x_i^{(s)}(t_1^{(s)}))/2 = & -a_i^{(s-1,s)} \cdot (x_i^{(s-1)}(t_{N^{(s-1)}}^{(s-1)}) + x_i^{(s-1)}(t_1^{(s-1)}))/2 \\ & + \sum_{j \in R_i^{(s-1)}} \left(b_{ij}^{(s-1,s)} \cdot (x_j^{(s-1)}(t_{N^{(s-1)}}^{(s-1)}) + x_j^{(s-1)}(t_1^{(s-1)}))/2 \right) \\ & + \sum_{j \in R_i^{(s)}} \left(b_{ij}^{(s)} \cdot (x_j^{(s)}(t_{N^{(s)}}^{(s)}) + x_j^{(s)}(t_1^{(s)}))/2 \right) \end{aligned} \tag{13}$$

Formula (13) will hold sufficiently if below conditions are true

$$x_i^{(s)}(t_{N^{(s)}}^{(s)}) = -a_i^{(s-1,s)} \cdot x_i^{(s-1)}(t_{N^{(s-1)}}^{(s-1)}) + \sum_{j \in R_i^{(s-1)}} \left(b_{ij}^{(s-1,s)} \cdot x_j^{(s-1)}(t_{N^{(s-1)}}^{(s-1)}) \right) + \sum_{j \in R_i^{(s)}} \left(b_{ij}^{(s)} \cdot x_j^{(s)}(t_{N^{(s)}}^{(s)}) \right) \tag{14}$$

$$x_i^{(s)}(t_1^{(s)}) = -a_i^{(s-1,s)} \cdot x_i^{(s-1)}(t_1^{(s-1)}) + \sum_{j \in R_i^{(s-1)}} \left(b_{ij}^{(s-1,s)} \cdot x_j^{(s-1)}(t_1^{(s-1)}) \right) + \sum_{j \in R_i^{(s)}} \left(b_{ij}^{(s)} \cdot x_j^{(s)}(t_1^{(s)}) \right) \tag{15}$$

In other words, by enforcing (12), (14) and (15), the linearity of gene evolving at the middle, starting and ending time of a stage will be ensured. In general, if we define λ to be a fraction factor that is associated with the time t_λ in a stage e.g. $t_\lambda^{(s-1)} = t_1^{(s-1)} + \lambda \cdot (t_{N^{(s-1)}}^{(s-1)} - t_1^{(s-1)})$ and $t_\lambda^{(s)} = t_1^{(s)} + \lambda \cdot (t_{N^{(s)}}^{(s)} - t_1^{(s)})$ for stage *s*-1 and *s*. According to the intra-stage steady-rate assumption, we have

$$x_i^{(s-1)}(t_\lambda^{(s-1)}) = x_i^{(s-1)}(t_1^{(s-1)}) + \lambda \cdot (x_i^{(s-1)}(t_{N^{(s-1)}}^{(s-1)}) - x_i^{(s-1)}(t_1^{(s-1)})) \tag{16}$$

$$x_i^{(s)}(t_\lambda^{(s)}) = x_i^{(s)}(t_1^{(s)}) + \lambda \cdot (x_i^{(s)}(t_{N^{(s)}}^{(s)}) - x_i^{(s)}(t_1^{(s)})) \tag{17}$$

Similar relationships hold for $x_j^{(s-1)}(t_\lambda^{(s-1)})$ and $x_j^{(s)}(t_\lambda^{(s)})$ in (14) and (15). By combining (16), (17) with (14) and (15), we get:

$$x_i^{(s)}(t_\lambda^{(s)}) = -a_i^{(s-1,s)} \cdot x_i^{(s-1)}(t_\lambda^{(s-1)}) + \sum_{j \in R_i^{(s-1)}} \left(b_{ij}^{(s-1,s)} \cdot x_j^{(s-1)}(t_\lambda^{(s-1)}) \right) + \sum_{j \in R_i^{(s)}} \left(b_{ij}^{(s)} \cdot x_j^{(s)}(t_\lambda^{(s)}) \right) \tag{18}$$

in which λ is a real number. Thus, equation (18) describes the inter-stage dynamical GRN. Note that (1) describes intra-stage dynamical GRN for each stage *s*. Also, (18) is referred as the model function of the dynamic cascaded method (DCM). Multiple equations of (18) can be generated on different settings of λ . The model coefficients can be solved by a linear regression approach such as LASSO.

Section two: Gene evolving trend analysis

Gene evolving trend analysis is to determine the gene evolving trend in each stage. Suppose there are *S* stages in a biological process. Let $t_1^{(s)}$ and $t_{N^{(s)}}^{(s)}$ are the starting and ending times of stage s, $x(t_1^{(s)})$ and $x(t_{N^{(s)}}^{(s)})$ are the gene expressions at the corresponding times. According to the continuity assumption, the gene expression profiles of two neighbouring stages should be continuous, i.e. $x(t_{N^{(s-1)}}^{(s-1)})$ should be equal to $x(t_1^{(s)})$. In practice, there could be error at the connecting point of two stages, i.e. $x(t_1^{(s)}) - x(t_{N^{(s-1)}}^{(s-1)})$. The overall connecting error can thus be defined as the L1-norm of all the individual connecting errors: $\sum_{s=2}^{S} \left| x(t_{N^{(s-1)}}^{(s-1)}) - x(t_1^{(s)}) \right|$, as shown in Figure S4 (a).

On the other hand, according to intra-stage steady-rate assumption, gene evolves linearly in a stage, so $x(t_1)$ and $x(t_N)$ should be corresponding to either the minimal or the maximal gene expression of the stage. With the sample-based data of a gene, we can obtain a set of minimal and maximal expressions of each individual stage, $\{x_{\min}^{(s)}, x_{\max}^{(s)} \,|\, s \in [1, S]\}$. Gene evolving trend analysis is to find an optimal path of travelling from the first stage to the last stage that can minimize the overall connecting error, as shown in Figure S4 (b). Once $x(t_1)$ and $x(t_N)$ are assigned to the minimal or maximal value, the gene evolving trend

is determined. For example, if $x(t_1)$ is the minimal value, and $x(t_N)$ is the maximal one, then the gene evolving trend is ascending, or vice versa.

The codes are downloadable at: http://www.comp.hkbu.edu.hk/~hlzhu/NAR_codes.html

Supplementary Table

Table S1. Enrichment analysis result of dynamic cascaded modelling on the HCC progression

	**Normal**	**Cirrhotic**	**Dysplastic**	**Early HCC**	**Advance HCC**
**Proportion of known interactions**					
PER =0.01	46.2	61.5	42.3	50.0	46.2
PER =0.02	36.5	42.3	44.2	42.3	36.5
PER =0.03	35.4	39.2	38.0	38.0	35.4
PER =0.04	31.4	36.2	33.3	37.1	36.2
PER =0.05	28.8	36.4	31.1	34.1	35.6
PER =0.10	25.3	31.3	28.7	27.8	30.0
PER =0.25	24.9	26.8	26.5	25.2	28.4
**Enrichment of known interactions**					
PER =0.01	100.7	167.5	83.9	117.4	100.7
PER =0.02	58.9	83.9	92.3	83.9	58.9
PER =0.03	54.1	70.6	65.1	65.1	54.1
PER =0.04	36.6	57.3	44.9	61.5	57.3
PER =0.05	25.2	58.1	35.0	48.2	54.8
PER =0.10	9.9	36.2	24.7	20.9	30.3
PER =0.25	8.2	16.5	15.2	9.3	23.4
**P-values of significance tests**					
PER =0.01	0.0179	0.0001	0.0463	0.0059	0.0179
PER =0.02	0.0427	0.0048	0.0021	0.0048	0.0427
PER =0.03	0.0208	0.0031	0.0061	0.0061	0.0208
PER =0.04	0.0629	0.0049	0.0247	0.0027	0.0049
PER =0.05	0.1421	0.0014	0.0454	0.0072	0.0025
PER =0.10	0.3926	0.0035	0.0410	0.0794	0.0130
PER =0.25	0.2597	0.0268	0.0408	0.2020	0.0021

Supplementary Figures

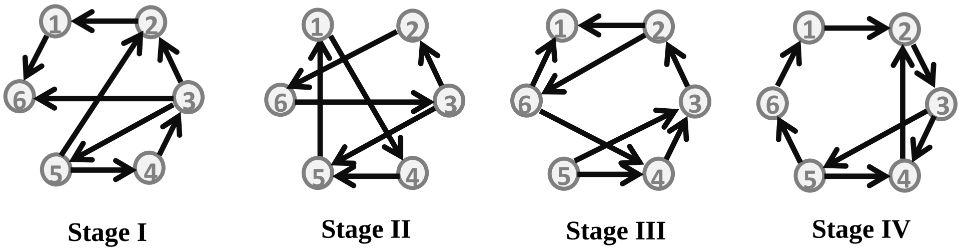

Figure S1. The topological structure of the *in silico* gene regulatory networks in four consecutive stages. The nodes represent genes and the arrows represent the regulatory relationships.

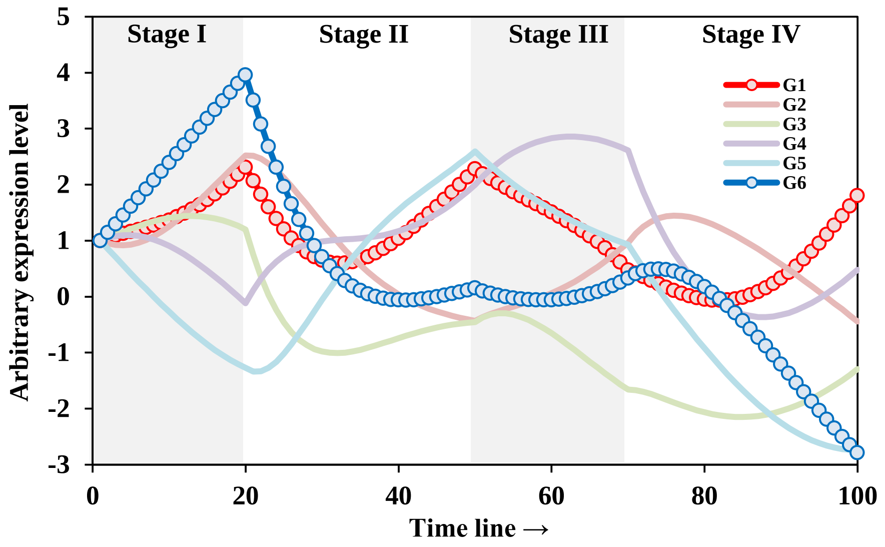

Figure S2. The time-course gene profiles generated from the *in silico* networks. The initial expression levels of all genes were set to 1.0 at the beginning of the process. The time spans of the four stages were set to 20, 30, 20 and 30, respectively.

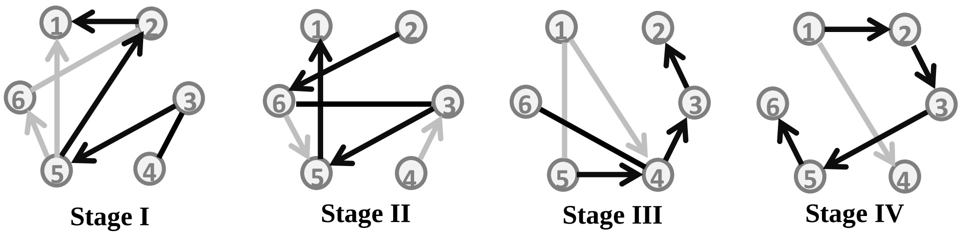

Figure S3. The GRNs reconstructed from the sample-based data by DCM with 10-fold cross validation. The edges in solid black are correct predictions, while the edges in grey are wrong predictions.

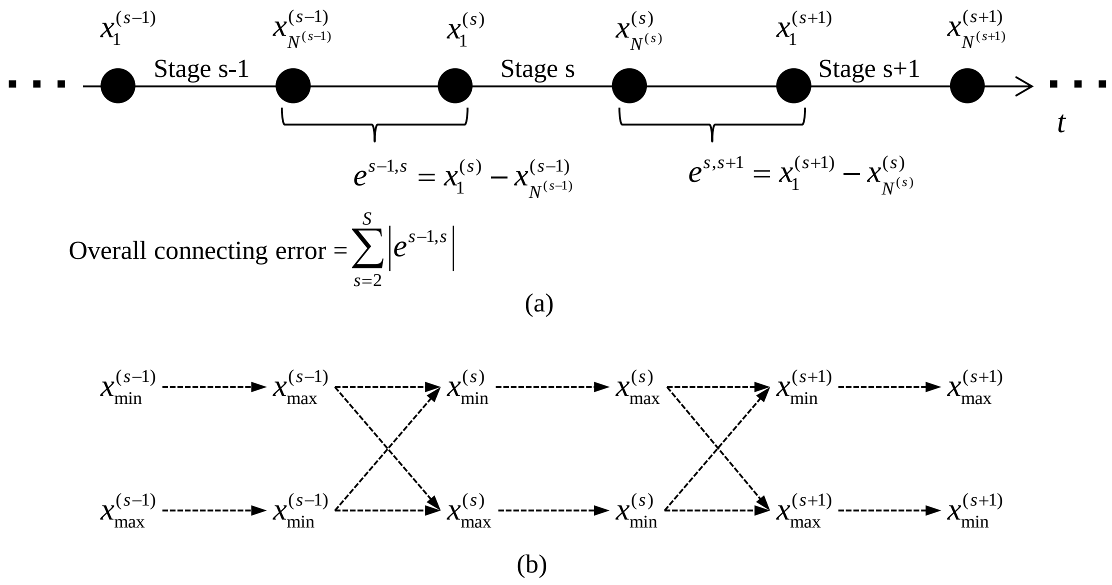

Figure S4. (a) Definition of connecting error in between two neighbouring stages. The arrow shows the evolving direction of a biological process, the white circle represents the starting point of a stage and the black circle represents the ending point. The overall connecting error is defined as the L1-norm of the individual connecting errors. (b) Possible traveling paths in gene evolving trend analysis. All possible traveling paths are shown with dash lines. Gene evolving trend analysis can be done by finding the optimal path of travelling from the first stage to the last stage that can minimize the overall connecting error.

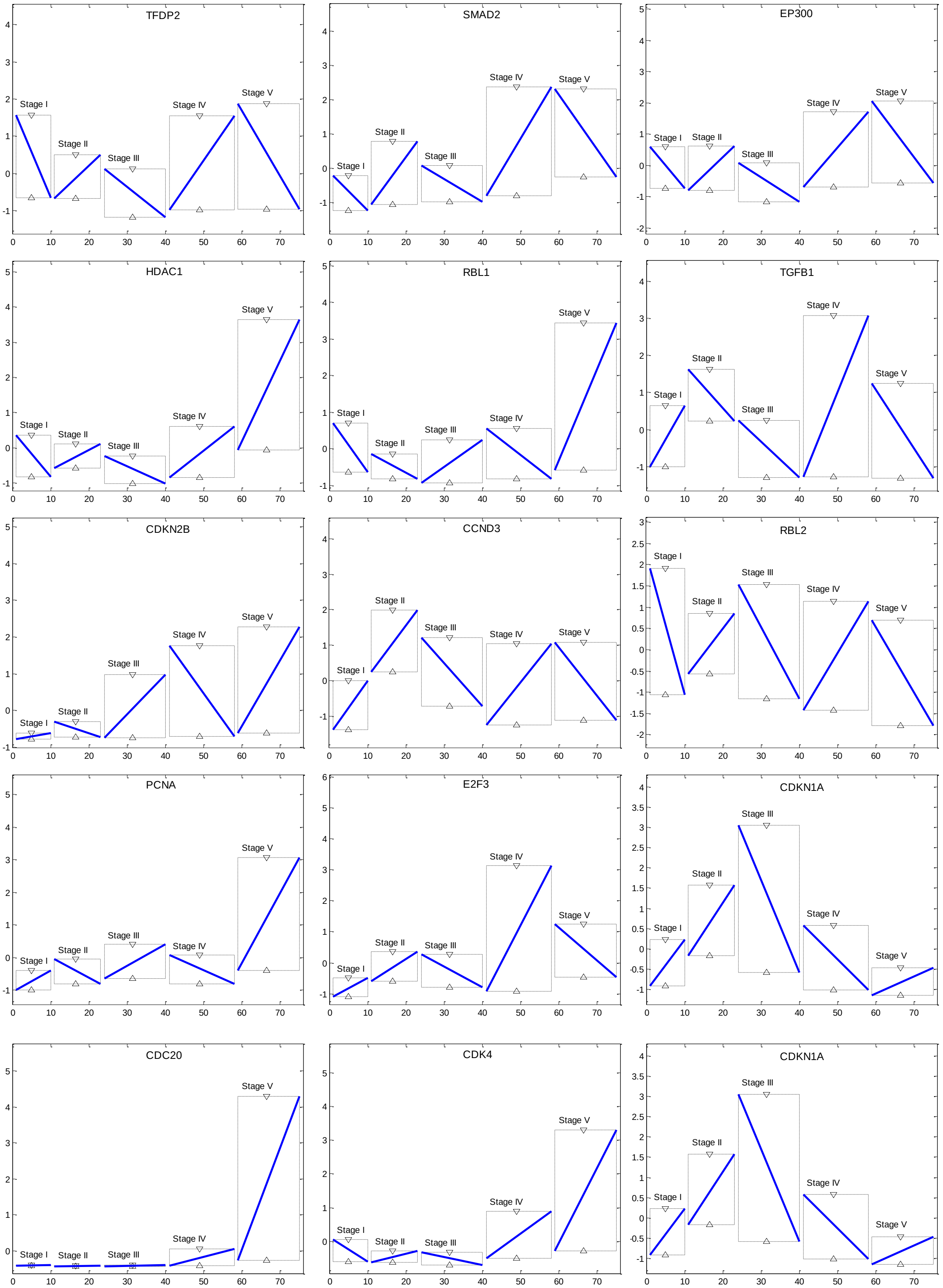
TFDP2
SMAD2
EP300
HDAC1
RBL1
TGFB1
CDKN2B
CCND3
RBL2
PCNA
E2F3
CDKN1A
CDC20
CDK4
CDKN1A
Stage I
Stage II
Stage III
Stage IV
Stage V

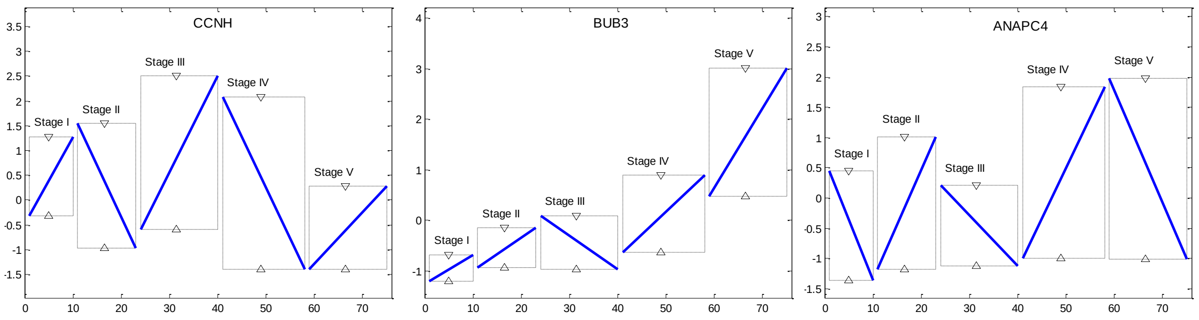

Figure S5. The stage-wise gene evolving trends of some hub genes. X-axis represents the gene evolving direction of cancer progression along five consecutive stages (I: normal, II: cirrhotic, III: dysplastic, IV: early HCC, and V: advance HCC), Y-axis represents the level of gene expression. The minimal and maximal gene expressions of each stage are marked with the symbols of "Δ" and "∇". The gene evolving trends of different stages are shown with solid blue lines.
